# Increasing Adhesive Layer Thickness Reduces Ultimate Load Capacity in Double-Lap CFRP-to-Steel Joints

**DOI:** 10.3390/ma19194244

**Published:** 2026-10-07

**Authors:** Baishun Xu, Yongjiu Qian, Chuan Xiao, Miao Zhang

**Affiliations:** 1 School of Civil Engineering and Architecture, Suqian University, Suqian 223800, China; xubaishun@squ.edu.cn; 2 School of Civil Engineering, Southwest Jiaotong University, Chengdu 610031, China; 3 Department of Transportation and Municipal Engineering, Sichuan University of Architectural Technology, Chengdu 610399, China

**Keywords:** CFRP-to-steel joint, ultimate load capacity, interfacial shear stress, bond–slip constitutive, adhesive layer thickness

## Abstract

Carbon fiber-reinforced polymer (CFRP) strengthening relies on effective load transfer across the bonded interface, yet the influence of adhesive layer thickness remains incompletely quantified. Twelve double-lap CFRP-to-steel joints with adhesive layer thicknesses of 0.5, 1.0, 1.5 and 2.0 mm were tested under displacement-controlled tension. CFRP strain measurements were used to determine interfacial shear stress distributions and local bond–slip responses. All specimens failed abruptly by mixed debonding at the CFRP–adhesive and steel–adhesive interfaces. Increasing the adhesive layer thickness from 0.5 to 2.0 mm reduced the mean ultimate load capacity from 126.24 to 79.42 kN. The mean nominal interfacial shear stress decreased from 8.42 to 5.29 MPa, and the fitted interfacial stiffness decreased by 45.4%. At higher load levels, shear stress concentrated near both bond ends and remained greater at the loaded end. The measured bond–slip curves showed no descending branch, consistent with the brittle response of the low-ductility adhesive. A thickness-dependent analytical model reduced the group-level mean absolute percentage error from 19.0% for the Xia & Teng model to 6.9%. It also reduced the root-mean-square error from 18.7 to 7.3 kN. Within the tested material system and thickness range, thinner adhesive layers therefore provided stiffer load transfer and higher ultimate load capacity.

## 1. Introduction

Carbon fiber-reinforced polymer (CFRP) sheets and plates are widely used to strengthen civil infrastructure [1,2,3]. The performance of these systems is often governed by the bonded CFRP-to-steel interface, where debonding is a common failure mode [4,5,6]. Over the past decade, extensive research has been conducted on the interfacial bond behavior of CFRP-to-steel bonded joints [7,8,9]. To ensure the effectiveness of the CFRP-to-steel interface, it is essential to establish a reliable bond–slip constitutive model, thoroughly understand the interfacial debonding process, and develop a dependable model for predicting the load capacity of the interface [10,11,12].

Numerous factors, including curing temperature, marine atmospheric environment, adhesive type, adhesive layer thickness, and testing conditions, affect the bond strength of CFRP-to-steel interfaces, and extensive research has been conducted on this topic [13]. As a viscoelastic material, the mechanical properties of the epoxy adhesive are highly dependent on the ambient temperature. Chandrathilaka et al. [14] prepared 82 CFRP/steel double-lap joints and conducted static tensile tests after elevated-temperature curing, revealing that such curing reduces the bond strength. He et al. [15] examined the mechanical behavior of CFRP–steel lap joints bonded with Sika30 and Araldite2015 adhesives at three temperatures (23 °C, *T*g − 15 °C, and *T*g + 15 °C) and found that the bond–slip curve transitioned from a trapezoidal shape to a triangular shape with increasing temperature. In contrast, Guo et al. [16] investigated single-lap bonded joints fabricated with Araldite2015 structural adhesive and tested at 25 °C, 0 °C, −20 °C, and −40 °C. They reported that both the interfacial shear stiffness and the interfacial fracture energy decreased at low temperatures.

Structural adhesives for CFRP-to-steel bonding can be broadly grouped by ductility. High-ductility adhesives commonly produce trapezoidal bond–slip curves, whereas low-ductility adhesives produce approximately triangular curves. Ke et al. [5] developed J-69E, an epoxy-film adhesive supported by a nylon-fabric carrier, and reported high bond strength, ductility and resistance to elevated temperature. Fernando et al. [17] derived a full-range analytical solution for joints with ductile nonlinear adhesives. Their analysis indicated that bond strength is governed primarily by interfacial fracture energy rather than adhesive tensile strength, and that the resulting bond–slip curve is approximately trapezoidal.

Beyond curing conditions, the performance of bonded CFRP-to-steel joints is governed by adhesive selection, adhesive layer thickness, joint configuration, and loading regime. Borrie et al. [1] identified epoxy as the predominant adhesive for FRP-to-steel bonding, while also listing polyester, phenolic, and polyurethane as alternative resin systems. They described single- and double-lap shear tests as common joint configurations and emphasized that adhesive properties and layer thickness affect stress transfer, bond strength, and failure mode. Thin, uniform adhesive layers are generally preferred, whereas increasing thickness can reduce bond strength and shift failure from cohesive debonding toward CFRP delamination [1]. Under cyclic loading, Papa et al. [18] combined CFRP coupon tests, single-lap joint tests, and numerical simulations to investigate coupled composite and interfacial damage. They found that fatigue damage in the CFRP reduced joint fatigue life, particularly at relatively low fatigue load ranges. Zhang et al. [9] used double-shear specimens to investigate CFRP-strengthened corroded steel plates under fatigue loading. They reported progressive interfacial stiffness degradation and strong effects of corrosion and load level on fatigue life. Their proposed fatigue-life model achieved prediction errors below 8%. Collectively, these studies clarify the roles of adhesive type, joint configuration, cyclic damage, and corrosion. However, they do not systematically isolate adhesive layer thickness in double-lap CFRP plate-to-steel joints. Accordingly, the present study examines four adhesive layer thicknesses under monotonic tensile–shear loading and develops a thickness-dependent model for predicting ultimate load capacity.

To investigate the effect of adhesive layer thickness on the interfacial bond behavior of CFRP-strengthened steel plates, double-lap CFRP-to-steel specimens with different adhesive thicknesses were tested under tensile–shear loading. Based on the experimental results, the effects of adhesive thickness on the interfacial shear-stress distribution and bond–slip behavior were analyzed. The relationship of the maximum interfacial shear stress to the adhesive tensile strength and adhesive thickness was also investigated. Furthermore, an analytical model incorporating the effect of adhesive thickness was derived to predict the ultimate load capacity of the bonded interface. These findings can inform the selection of an appropriate adhesive layer thickness for CFRP strengthening of steel structures.

## 2. Experimental Section

### 2.1. Specimen Geometry and Material Properties

In this study, the CFRP plates (1.4 mm thick and 50 mm wide) and carbon fiber plate adhesive were supplied by Tianjin Carbon Technology Co., Ltd. (Tianjin, China). The adhesive was a two-component modified epoxy comprising Part A (resin) and Part B (hardener). Before application, Parts A and B were mixed at a mass ratio of 2:1. The mixture was then cured through a chemical reaction to develop the required bond strength. The steel plates were made of 8 mm thick Q345 steel and were manufactured by Jiangsu Donggang Steel Co., Ltd. (Suqian, China). The mechanical properties of the CFRP plates and adhesive were provided by their manufacturer, whereas those of the steel plates were determined experimentally in accordance with the relevant standard [19]. The resulting mechanical properties of all materials are summarized in Table 1.

**Figure 1:**
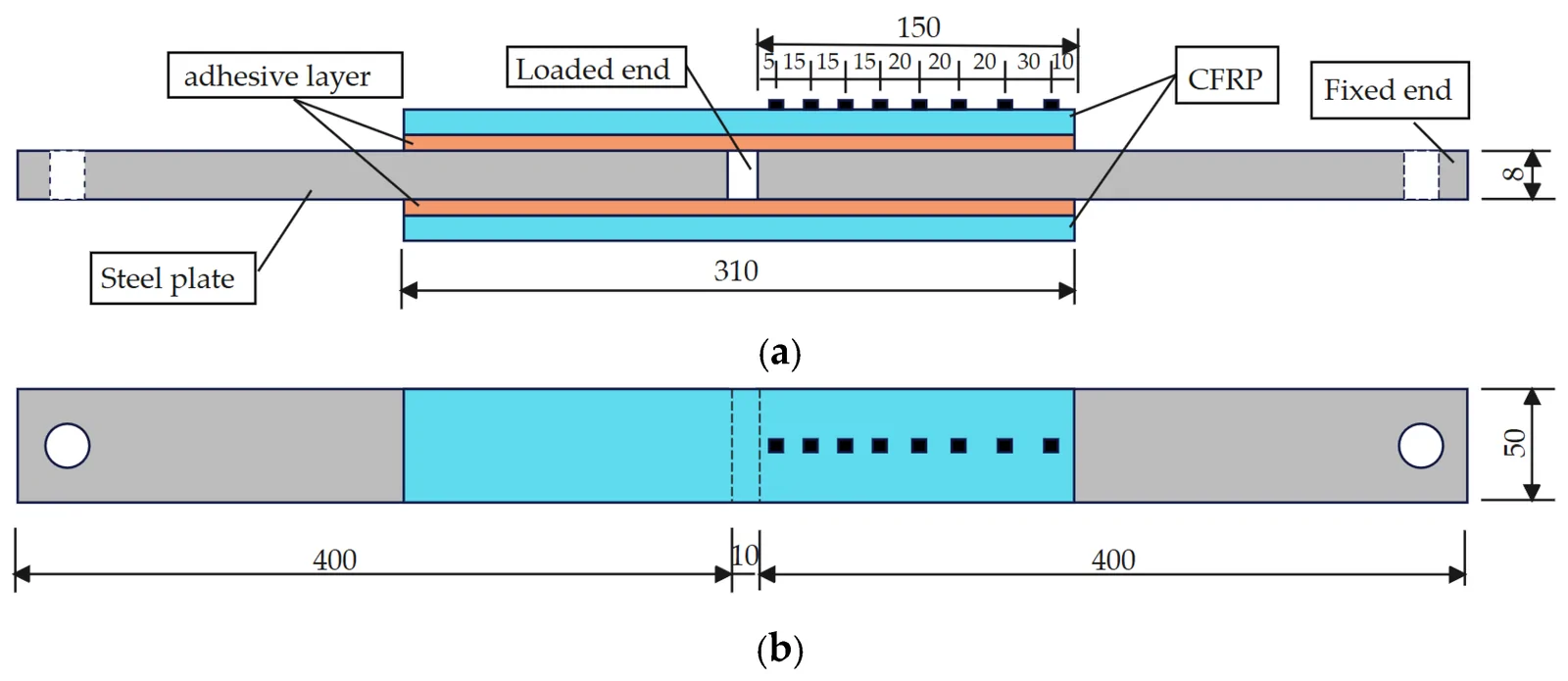
The CFRP–steel double-lap joint and the arrangement of strain gauges: (**a**) side view; (**b**) top view. All dimensions are in millimeters.

As shown in Figure 1, the specimens were designed as double-strap bonded joints. Each specimen was composed of two symmetrically arranged steel plates of identical dimensions, with each plate measuring 400 mm × 50 mm × 8 mm. The CFRP plates had a length of 310 mm, with a 10 mm gap reserved between the two steel plates. The bond length of the CFRP plate on each steel plate was 150 mm.

Twelve specimens were divided into four groups of three. The target adhesive layer thicknesses were 0.5, 1.0, 1.5 and 2.0 mm. Steel spheres with the corresponding diameters controlled the layer thickness during fabrication. Table 2 gives the specimen designations and measured thicknesses. Eight strain gauges were installed on each specimen at the locations shown in Figure 1.

Adhesive layer thickness was measured at four uniformly distributed points within each bonded region. The steel plate thickness, *t_s_*, was measured with a micrometer, and the nominal CFRP thickness, *t_c_*, was 1.4 mm. After fabrication, the total joint thickness was measured at each point. The local adhesive layer thickness, *t*_*ad*,*i*_, was calculated as

(1) $$t_{\mathrm{ad},i}=\frac{t_{\mathrm{total},i}-t_{s}-2t_{c}}{2}$$

where *t*_*total*,*i*_ is the total measured thickness at point *i*. Table 2 reports the four local measurements and their mean ± sample standard deviation (SD).

### 2.2. Specimen Fabrication and Experimental Procedure

**Figure 2:**
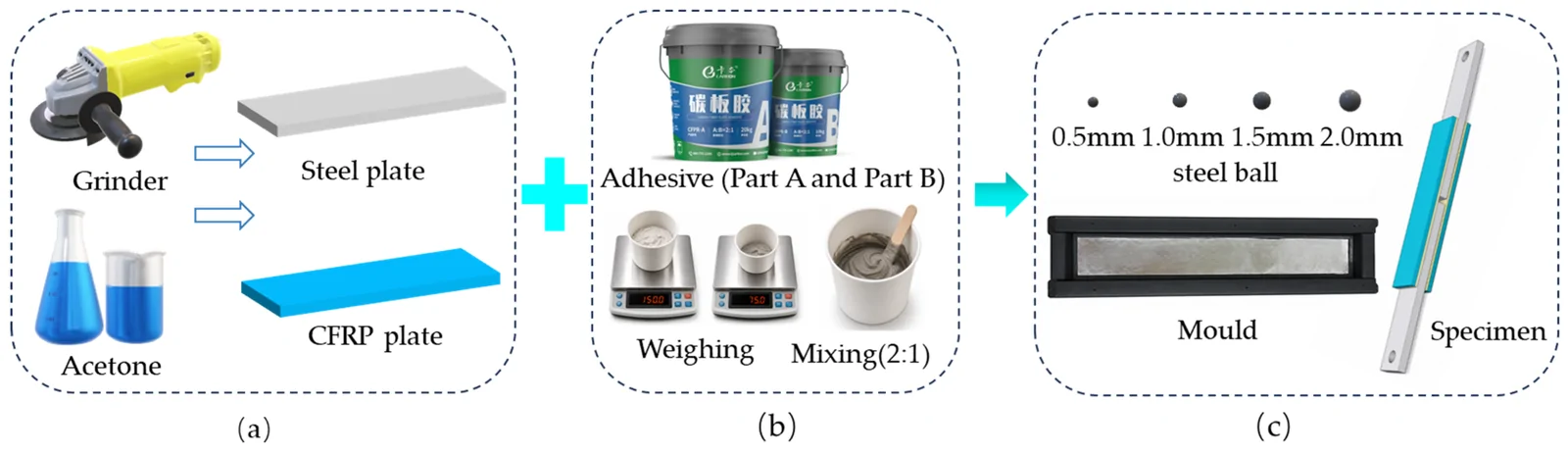
Fabrication of the CFRP–steel double-lap joint: (**a**) surface treatment; (**b**) adhesive producing; (**c**) specimen bonding.

The specimens were fabricated in six steps. First, the steel plates, CFRP plates and two-part epoxy were prepared. Second, the steel bonding surfaces were ground to re-move rust, cleaned with acetone-soaked cotton and marked according to Figure 1. Third, Parts A and B were weighed at a 2:1 mass ratio and mixed continuously for 5 min. Fourth, each steel plate was fixed on the mold, and the adhesive was applied with a brush. For each bonding face, four steel balls were placed within the designated bonded area to control the adhesive layer thickness. The CFRP plate was then bonded to the prepared face of the steel plate. A weight was placed over the bonded area of the CFRP plate to apply pressure and expel excess adhesive from the joint. The expelled adhesive was subsequently wiped away. Fifth, after curing for 24 h, the same procedure was used to bond the CFRP plate on the opposite face. The completed joints were cured for a further 7 days. Finally, strain gauges were attached at the prescribed locations. Figure 2 shows the principal fabrication stages.

**Figure 3:**
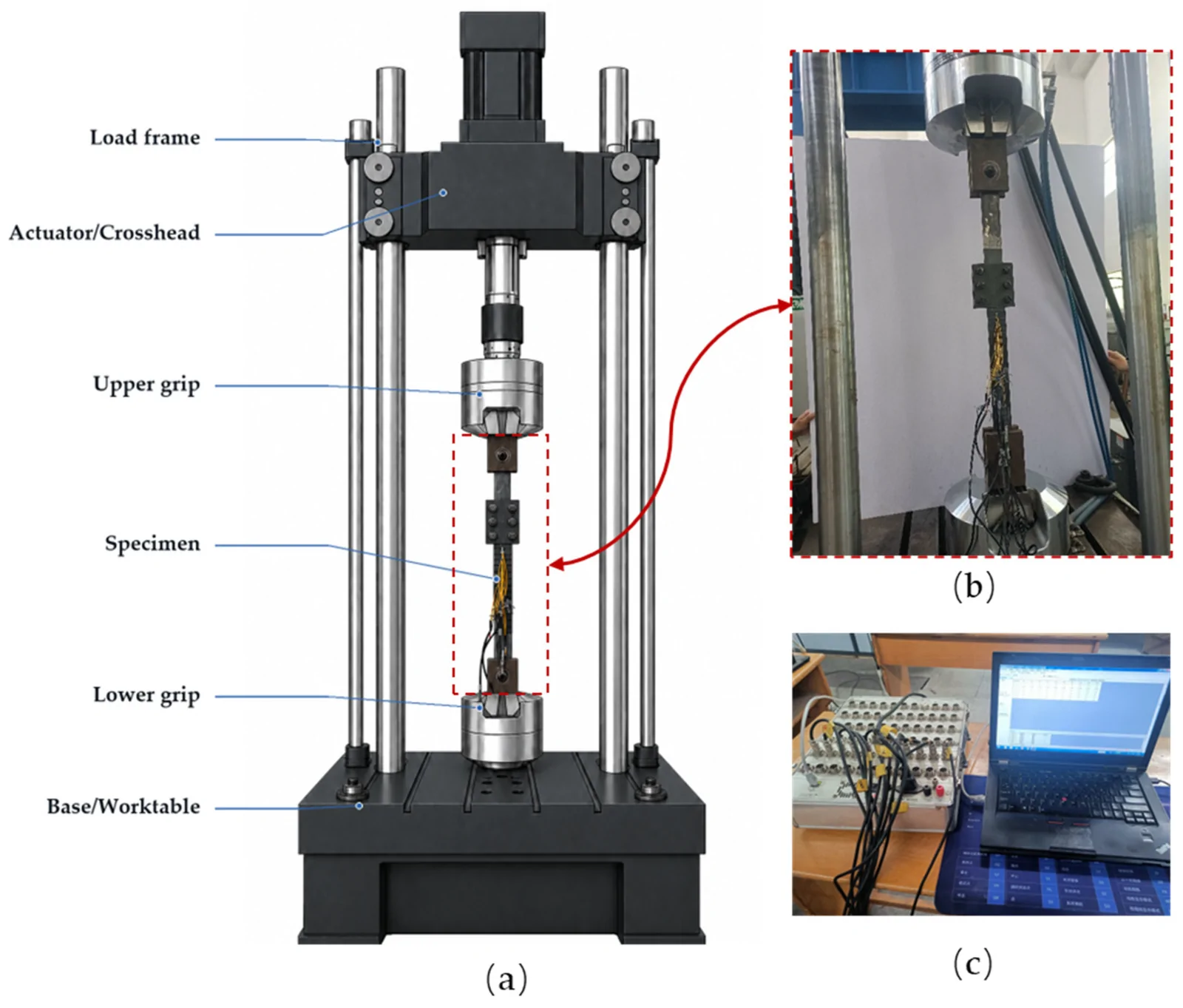
Tensile test of the CFRP–steel double-lap joint: (**a**) universal testing machine; (**b**) test specimen; (**c**) strain data acquisition system and computer.

Loading was applied using a 100 kN MTS electro-hydraulic servo testing machine under displacement control at a rate of 0.2 mm/min, as shown in Figure 3a. The specimen mounted in the testing machine is shown in Figure 3b. CFRP strains were recorded using a DH3816N static strain acquisition system, as shown in Figure 3c. Before formal loading, a preload was applied to verify the operation of the loading and data-acquisition systems and to take up any clearance between the specimen and the grips. To prevent premature debonding of the CFRP plate at the non-test end, a steel clamping fixture was installed at that end.

## 3. Results and Discussion

### 3.1. Failure Modes

**Figure 4:**
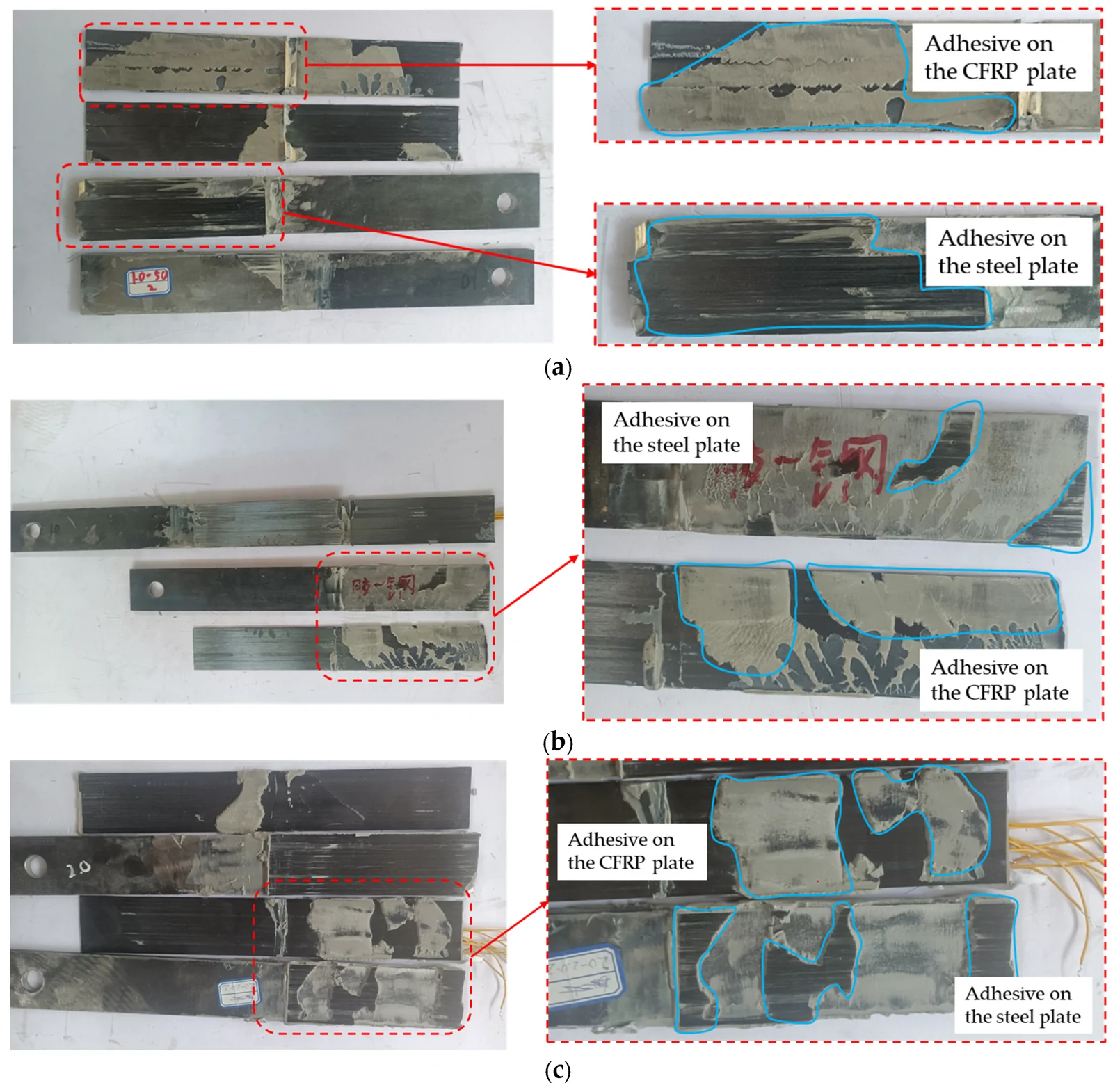
Typical failure mode: (**a**) specimen B-0.5-1; (**b**) specimen C-1.5-2; (**c**) specimen D-2.0-1.

The failure process observed across all specimen groups was largely similar. At the initial stage of loading, no cracking was visible as the load increased. As the applied load approached the ultimate load capacity, a sudden “bang” sound was heard, followed immediately by debonding failure of the specimen, and the load reading on the testing machine dropped to zero. CFRP-strengthened steel joints can fail by interfacial debonding, constituent fracture or plastic deformation [7]. Interfacial modes include separation at the steel–adhesive or adhesive–CFRP interface and cohesive cracking within the adhesive. Constituent failure includes CFRP delamination or tensile rupture, whereas the steel substrate may yield. The failure characteristics were essentially uniform among the groups, with a typical failure mode illustrated in Figure 4. Residual adhesive on both the CFRP and steel surfaces indicated mixed failure at the two adherend–adhesive interfaces and cohesive cracking within the adhesive layer.

The ultimate load capacities of all specimens are summarized in Table 3. Here, *P*_u_ denotes the ultimate load capacity, while *P*_u_ denotes the average ultimate load capacity for each experimental group. It can be observed from Table 3 that the smallest average ultimate load capacity, 79.42 kN, occurred at an adhesive thickness of 2.0 mm. In contrast, the largest average ultimate load capacity, 126.24 kN, was obtained at an adhesive thickness of 0.5 mm, which is 1.59 times that of the 2.0 mm case.

For the CFRP–steel double-lap joint, the nominal total bonded area on both sides of the interface was taken as *S*_nom_ = 2 × 150 × 50 mm^2^, based on the nominal specimen dimensions. The actual bonded dimensions may deviate slightly from the nominal dimensions because of fabrication and specimen preparation tolerances. Since the actual bonded area of each individual specimen was not measured in the present study, the interfacial stress reported herein is referred to as the mean nominal interfacial shear stress, calculated as *τ*_nom_ = *P*_u_/*S*_nom_. As the adhesive layer thickness increased from 0.5 mm to 2.0 mm, the mean nominal interfacial shear stress decreased from 8.42 MPa to 5.29 MPa. Therefore, both the average ultimate load capacity and the mean nominal interfacial shear stress decreased with increasing adhesive layer thickness.

### 3.2. Load–Displacement Relationships

**Figure 5:**
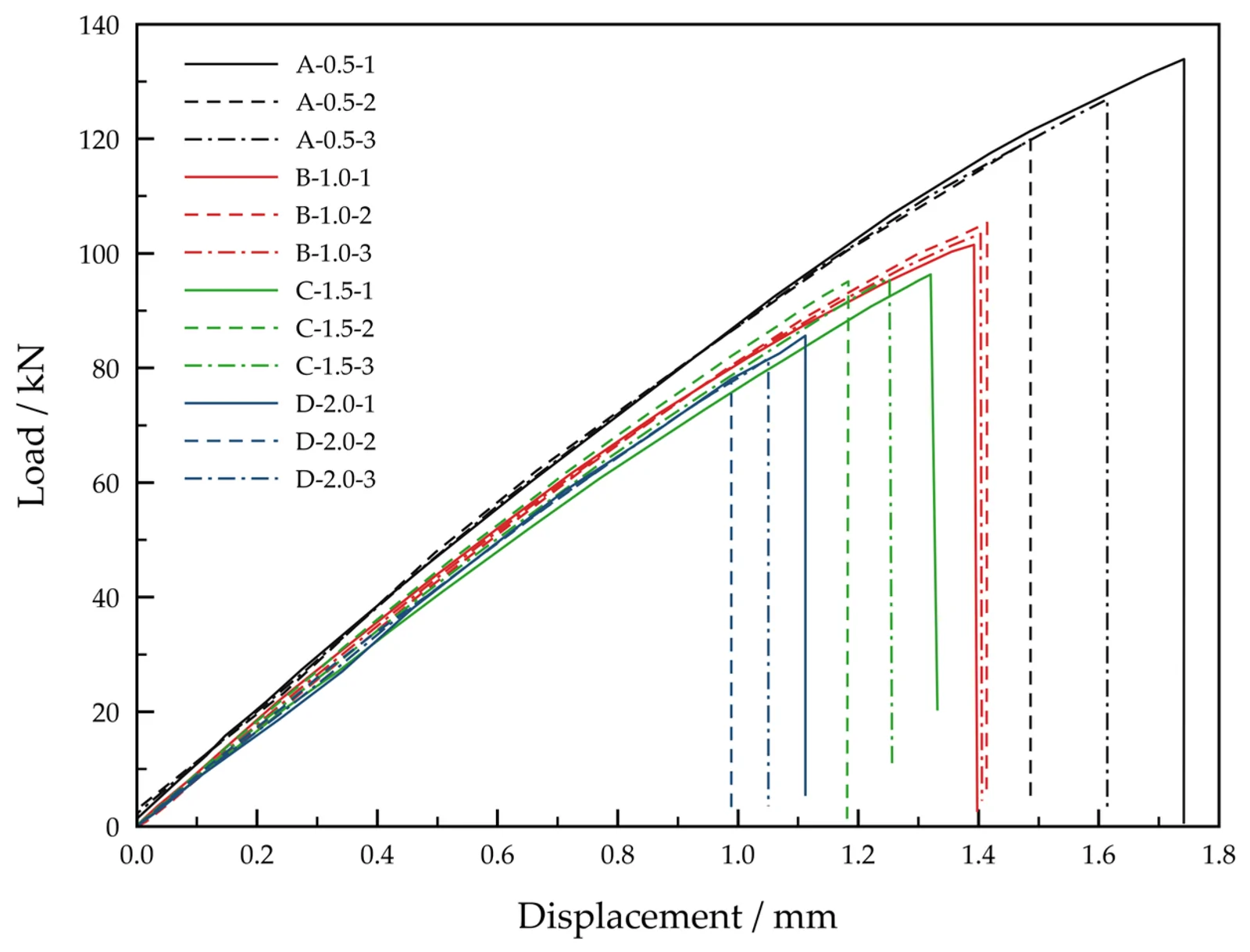
Load–displacement curve.

The load–displacement responses of individual specimens are presented in Figure 5. These curves can be broadly categorized as approximately linear with no obvious yield plateau. The mechanical response can be roughly divided into three typical stages: (1) Initial stiffness hardening stage: at the beginning of loading, the slope of the curve increases gradually with increasing load, reflecting a continuous enhancement of structural stiffness. (2) Linear elastic stage: the load and displacement exhibit a stable linear relationship, and the overall elastic modulus and stiffness of the specimen remain essentially constant. (3) Stiffness degradation stage: prior to failure, the curve slope begins to decrease. As interfacial debonding damage develops, the overall stiffness of the specimen progressively declines with increasing load, eventually leading to a sudden drop in load-carrying capacity due to brittle failure. Owing to the low ductility of the adhesive used, the stiffness degradation process is relatively short, and no distinct yielding is observed. The specimens display typical brittle failure characteristics, which are consistent with the failure process described.

### 3.3. CFRP Strain Distribution

**Figure 6:**
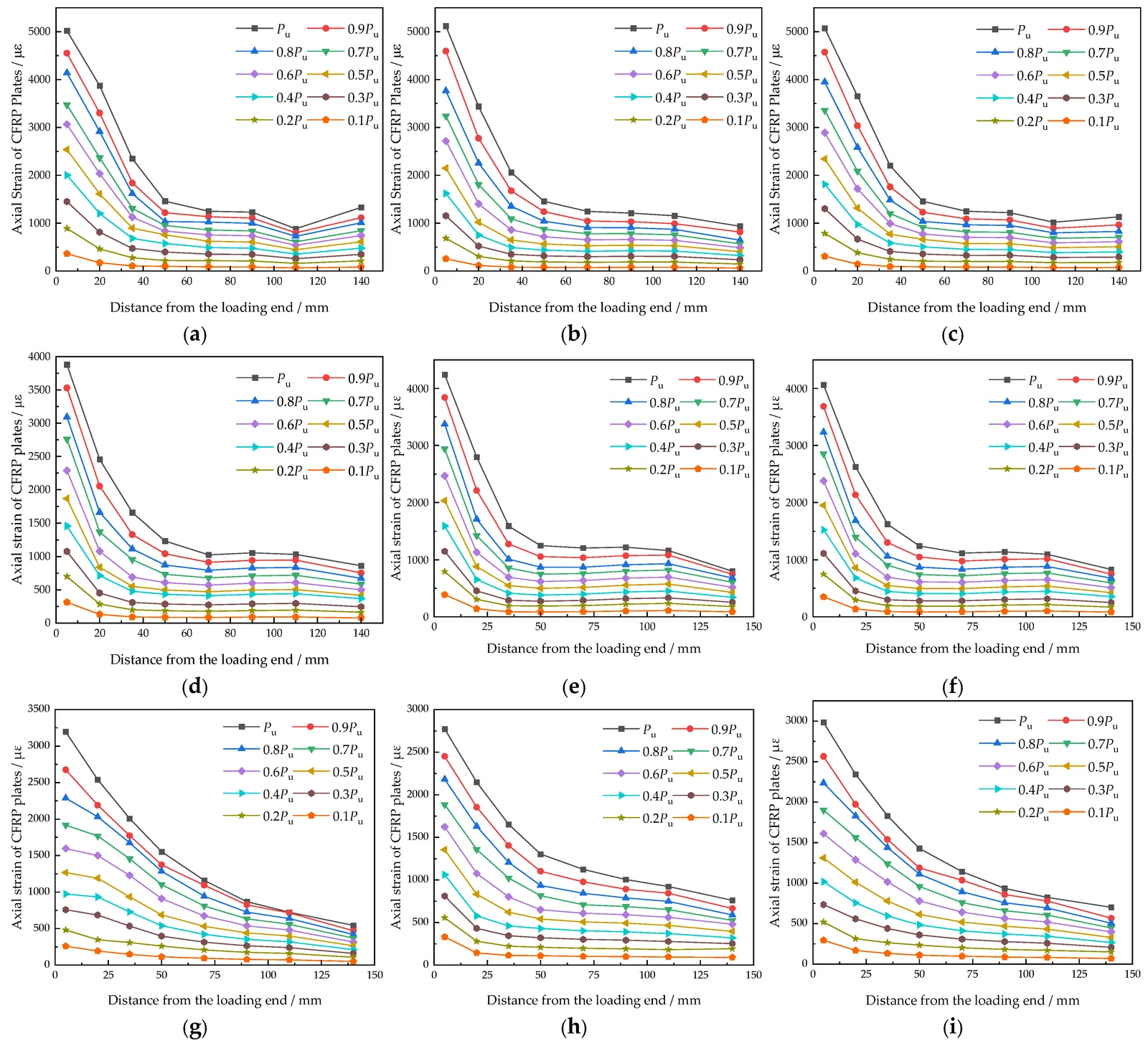

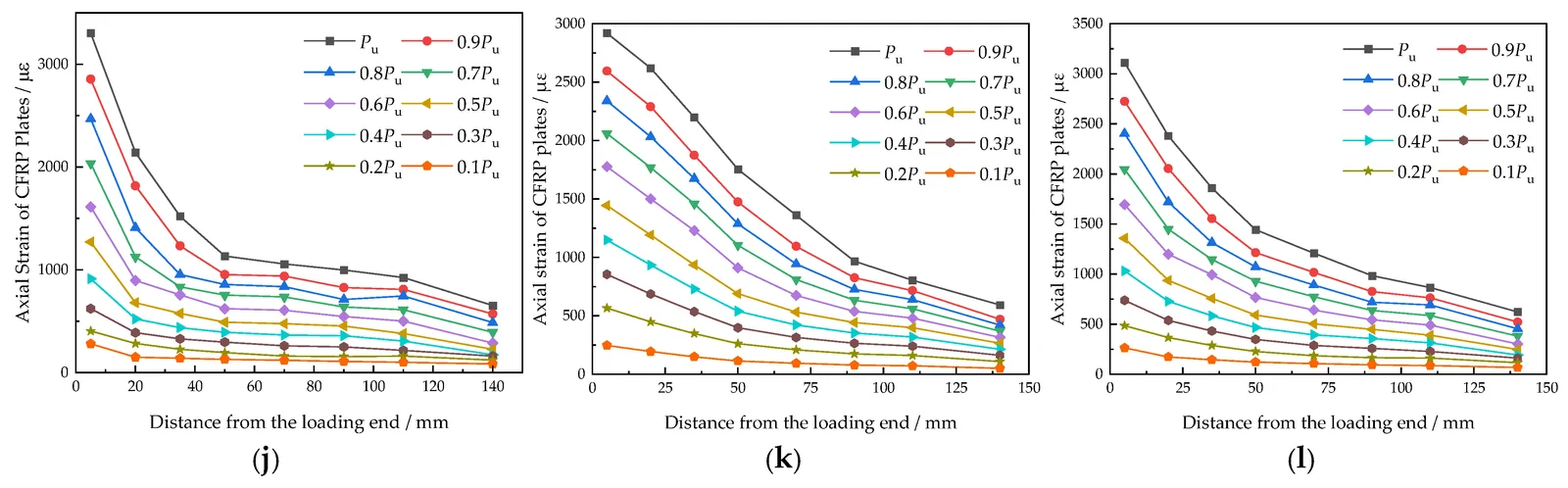
Axial CFRP strain distributions: (**a**) specimen A-0.5-1; (**b**) specimen A-0.5-2; (**c**) specimen A-0.5-3; (**d**) specimen B-1.0-1; (**e**) specimen B-1.0-2; (**f**) specimen B-1.0-3; (**g**) specimen C-1.5-1; (**h**) specimen C-1.5-2; (**i**) specimen C-1.5-3; (**j**) specimen D-2.0-1; (**k**) specimen D-2.0-2; (**l**) specimen D-2.0-3.

By compiling the strain data, the distribution curves of CFRP axial strain at different loading stages can be obtained. Figure 6 illustrates the axial strain distribution along the CFRP surface, where *P*_u_ represents the ultimate load. The evolution of the CFRP strain distribution reflects the stress state at the CFRP–steel interface throughout the entire loading process. As can be observed from the figures, the CFRP axial strain distribution curves for specimens with various adhesive thicknesses exhibit similar features, showing a general downward trend that follows an exponential form. Under each load level, the strain near the loaded end is relatively high, indicating that the CFRP plate is more actively engaged in load transfer in this region. With increasing load, the CFRP strain values increase accordingly, but the rate of increase diminishes with distance from the loaded end. At lower applied loads, only the portion of the CFRP plate near the loaded end participates in force transmission; as the load rises, the length of CFRP contributing to load transfer gradually increases.

The maximum peak strain typically occurs at the center of the specimen, which is attributable to the fact that debonding failure often initiates at this location and then propagates outward. During testing, a sudden surge in strain was observed in some specimens. This phenomenon is due to localized debonding or delamination of the CFRP plate as the load continues to increase. In such cases, the failed region no longer sustains stress, leading to a redistribution of interfacial stresses toward areas away from or adjacent to the failure site.

To provide a consistent operational measure of load-transfer activation, a CFRP strain of 200 με was adopted as the threshold. This strain corresponds to a CFRP stress of 36 MPa (200 με × 180 GPa), or approximately 1.4% (36/2625.5 × 100%) of its tensile strength, and therefore represents measurable engagement rather than material damage or full strength utilization. Based on this criterion, the entire instrumented bonded length became engaged at approximately 0.2*P*_u_, 0. *P*_u_, 0.4*P*_u_, and 0.5*P*_u_ for Groups A to D, respectively. The progressive delay in full-length engagement with increasing adhesive layer thickness is consistent with the lower interfacial stiffness of thicker adhesive layers, which reduces stress transfer to regions away from the loaded end at a given normalized load. Because the 200 με criterion is an operational threshold, the reported activation loads should be interpreted as threshold-dependent.

### 3.4. Interfacial Shear Stress

**Figure 7:**
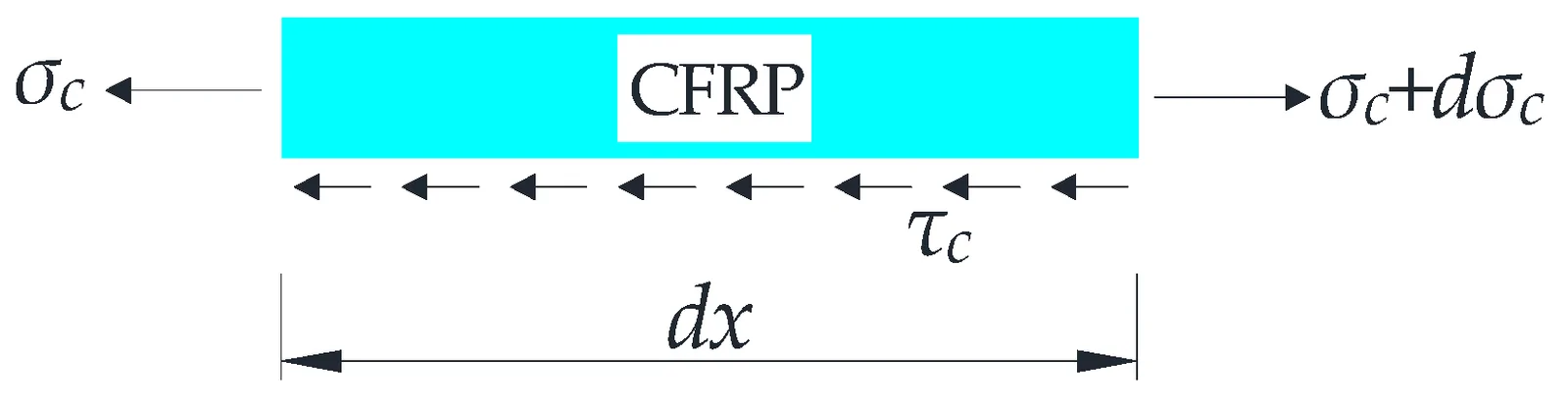
Force analysis of CFRP finite elements.

A differential element of the CFRP plate was taken for force analysis, as shown in Figure 7.

Based on the static equilibrium condition, the equilibrium equation is established as follows:

*σ_c_b_c_t_c_* + *τ_c_b_c_d_x_* = (*σ_c_* + *dσ_c_*)*b_c_t_c_* (2)

where *t_c_* represents the thickness of the CFRP plate, *b_c_* is the width of the CFRP plate, *σ_c_* denotes the normal stress in the CFRP plate, and *τ_c_* is the interfacial shear stress.

Let *E_c_* denote the elastic modulus of the CFRP plate and *ε_c_* the strain in the CFRP plate. Substituting *σ_c_* = *ε_c_E_c_* into Equation (2) and rearranging the terms gives

(3) $$\tau_{c}=E_{c}t_{c}\frac{d\varepsilon_{c}}{\mathrm{dx}}$$

Based on the principle of the finite difference method, the interfacial shear stress *τ*_*i*~*i*+1_ at the midpoint between the *i*-th and (*i* + 1)-th strain measuring points can be further expressed as

(4) $$\tau_{i\sim i+1}=E_{c}t_{c}\frac{d\varepsilon_{c}}{\mathrm{dx}}=\frac{E_{c}t_{c}\left(\varepsilon_{c,i+1}-\varepsilon_{c,i}\right)}{\Delta x}$$

where *E_c_* and *τ_c_* are the elastic modulus and thickness of the CFRP plate, respectively, *ε*_*c*,*i*_ and *ε*_*c*,*i*+1_ are the strains measured at the *i*-th and (*i* + 1)-th points, and Δ*x* is the distance between these two adjacent measuring points.

**Figure 8:**
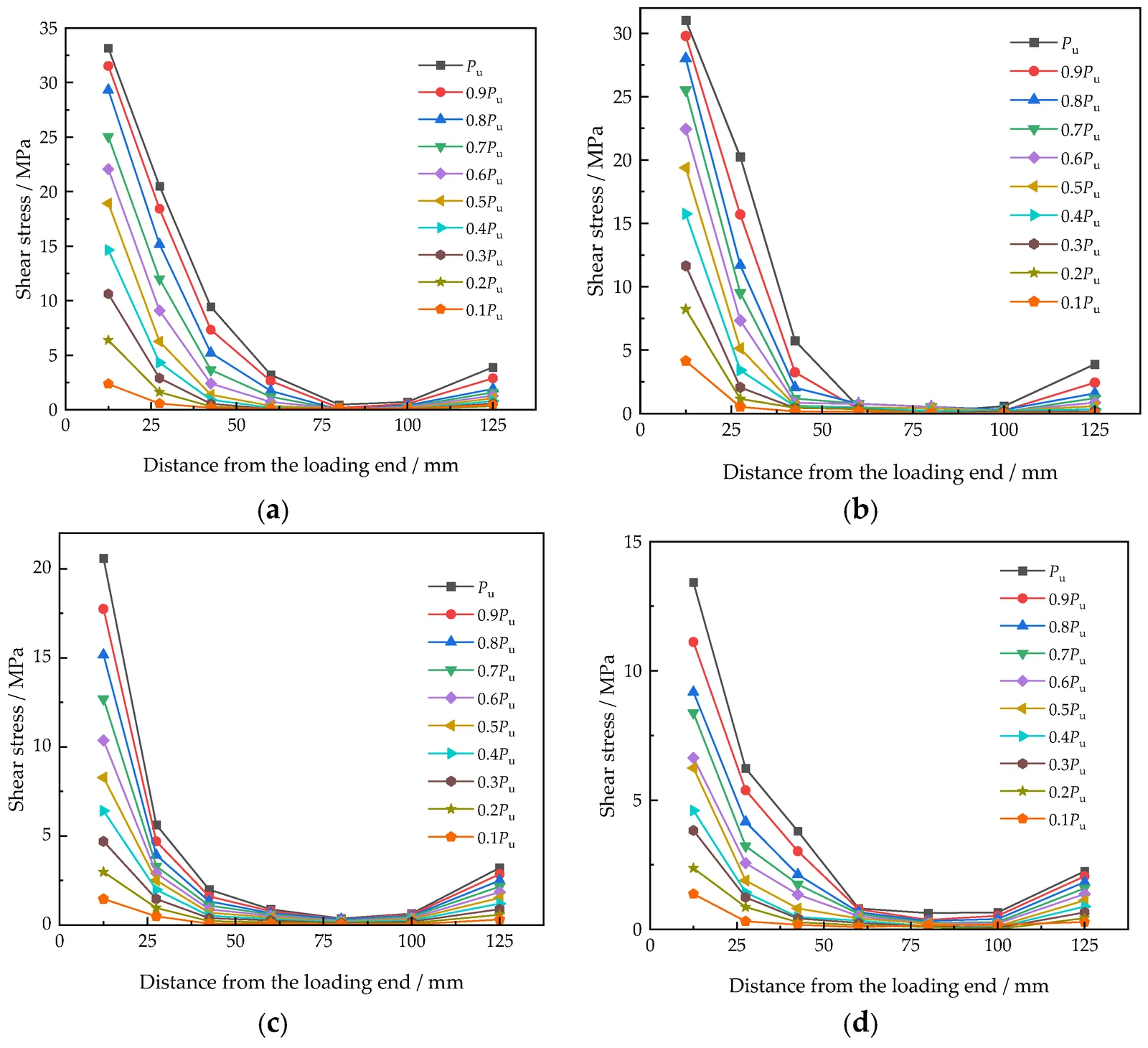
Interfacial shear stress: (**a**) specimen A-0.5-2; (**b**) specimen B-1.0-2; (**c**) specimen C-1.5-2; (**d**) specimen D-2.0-2.

The interfacial shear stress for each specimen can be determined from Equation (4). Figure 8 presents the distribution of interfacial shear stress during loading for one representative specimen selected from each group. As can be seen from Figure 8, the interfacial shear stress is unevenly distributed at various load levels, with the maximum value occurring at 12.5 mm from the loaded end. In other words, the closer to the loaded end, the higher the interfacial shear stress.

When the applied load is equal to or less than 0.2*P*_u_, the interfacial shear stress exhibits a decreasing trend from the loaded end toward the fixed end. When the load reaches or exceeds 0.3*P*_u_, the shear stress becomes relatively high at both ends and lower in the middle. Moreover, under the same load level, the shear stress at the loaded end is greater than that at the fixed end, indicating that the CFRP plate contributes more to load transfer in the region near the loaded end. For instance, for specimen A-0.5-2 at the ultimate load *P*_u_, the interfacial shear stress is 33.16 MPa at 12.5 mm from the loaded end, compared to 3.89 MPa at 125 mm from the loaded end.

A comparison among the specimen groups reveals that, at each load level, the specimen with an adhesive thickness of 0.5 mm exhibits higher interfacial shear stress at every measuring point than the other specimens. In other words, the interfacial shear stress at each measuring point decreases with increasing adhesive layer thickness. This further suggests that a smaller adhesive thickness leads to greater participation of the CFRP plate in load transfer, and consequently, a higher ultimate load capacity of the interface.

## 4. Bond–Slip Behavior and Ultimate Load Capacity

### 4.1. Bond–Slip Behavior

By numerically integrating the strain data on the CFRP plate, the slip *s_i_* at the midpoint between the *i*-th and (*i* + 1)-th strain measuring points can be obtained as

(5) $$s_{i}=\sum_{j=0}^{i-1}\frac{\left(\varepsilon_{c,j+1}+\varepsilon_{c,j}\right)}{2}\Delta x$$

**Figure 9:**
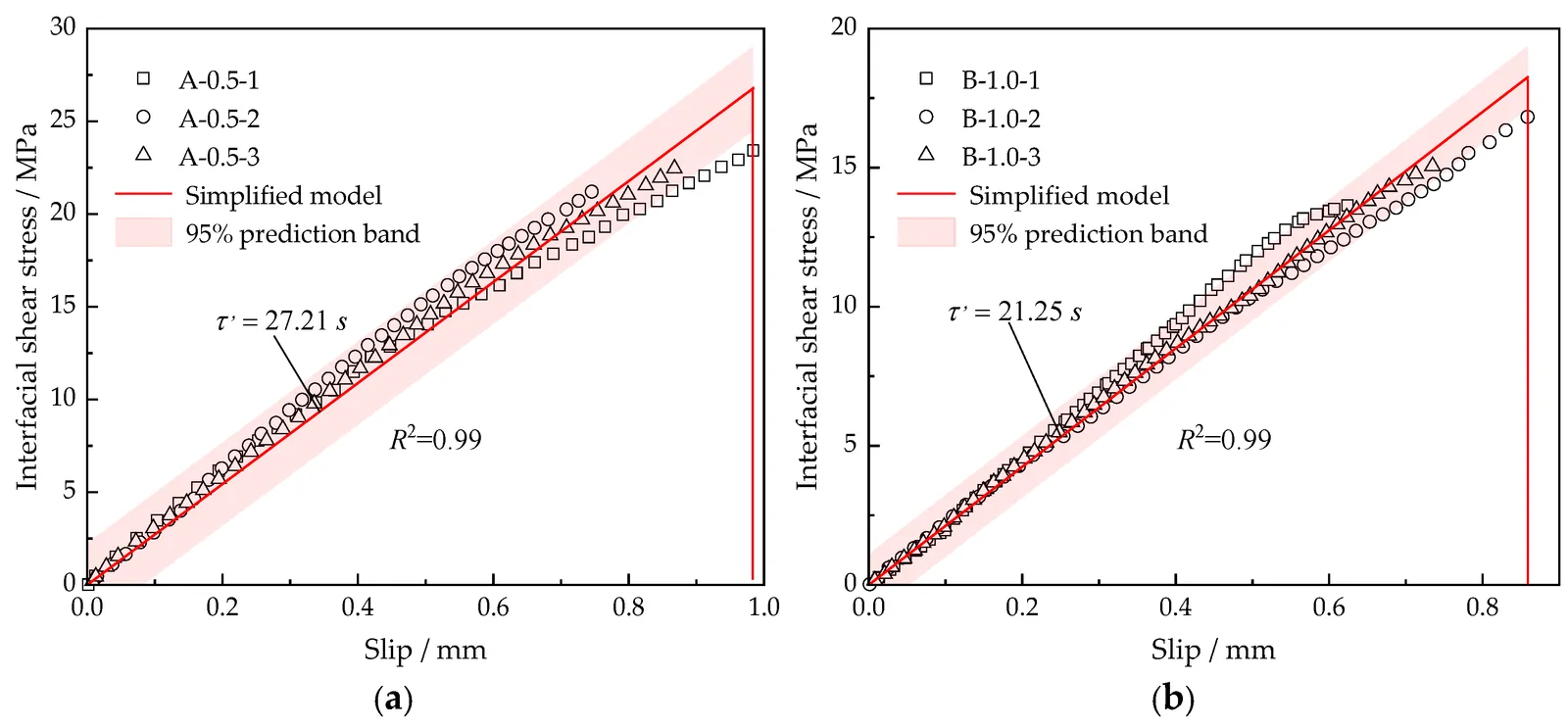

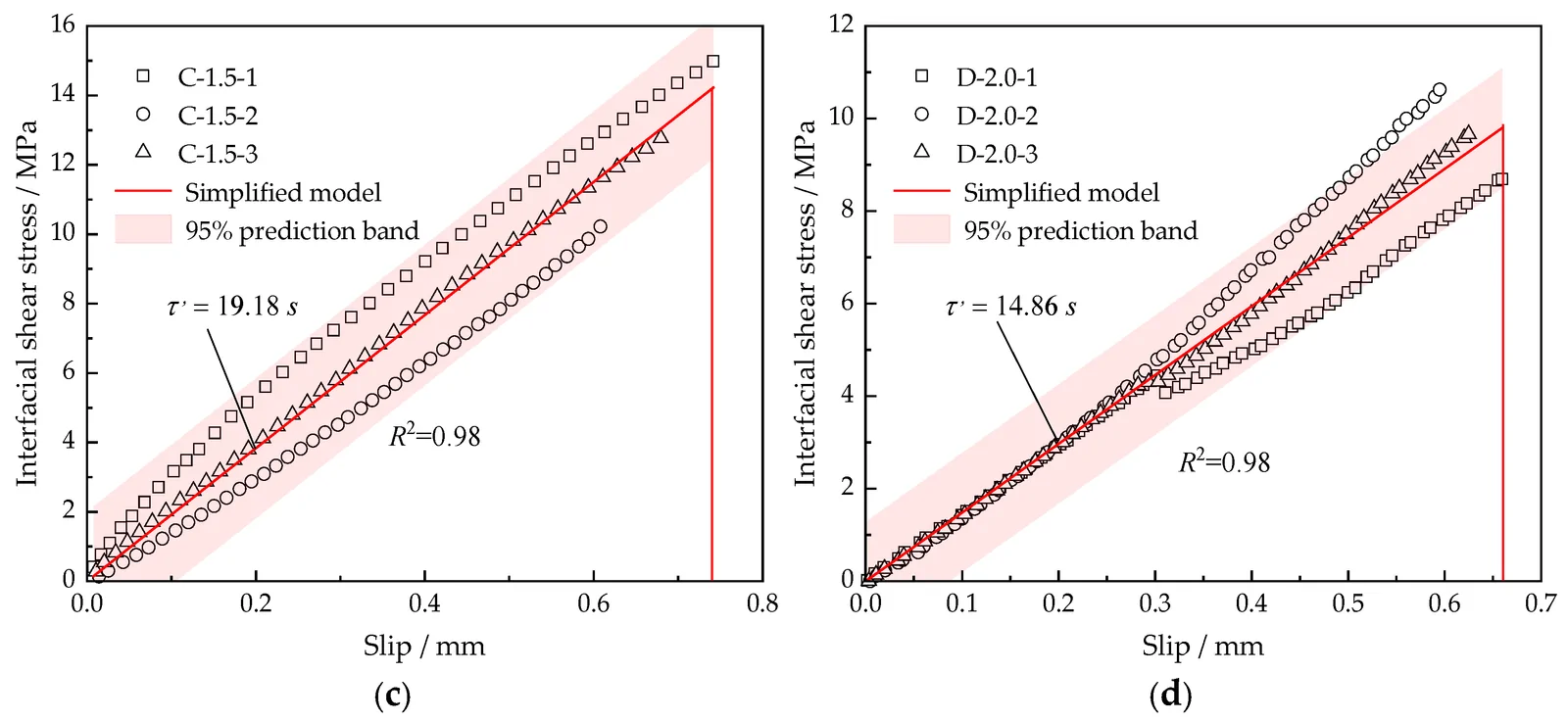
Bond–slip curves: (**a**) Group A specimens; (**b**) Group B specimens; (**c**) Group C specimens; (**d**) Group D specimens.

Using Equations (4) and (5), the interfacial shear stress and slip at each measuring point can be determined. In this test, the adhesive layer at the midpoint between the first and second measuring points (i.e., 12.5 mm from the loaded end) underwent the entire interfacial failure process; therefore, this location was selected to calculate the interfacial shear stress and slip, and the resulting bond–slip curves for each specimen group are plotted in Figure 9.

**Figure 10:**
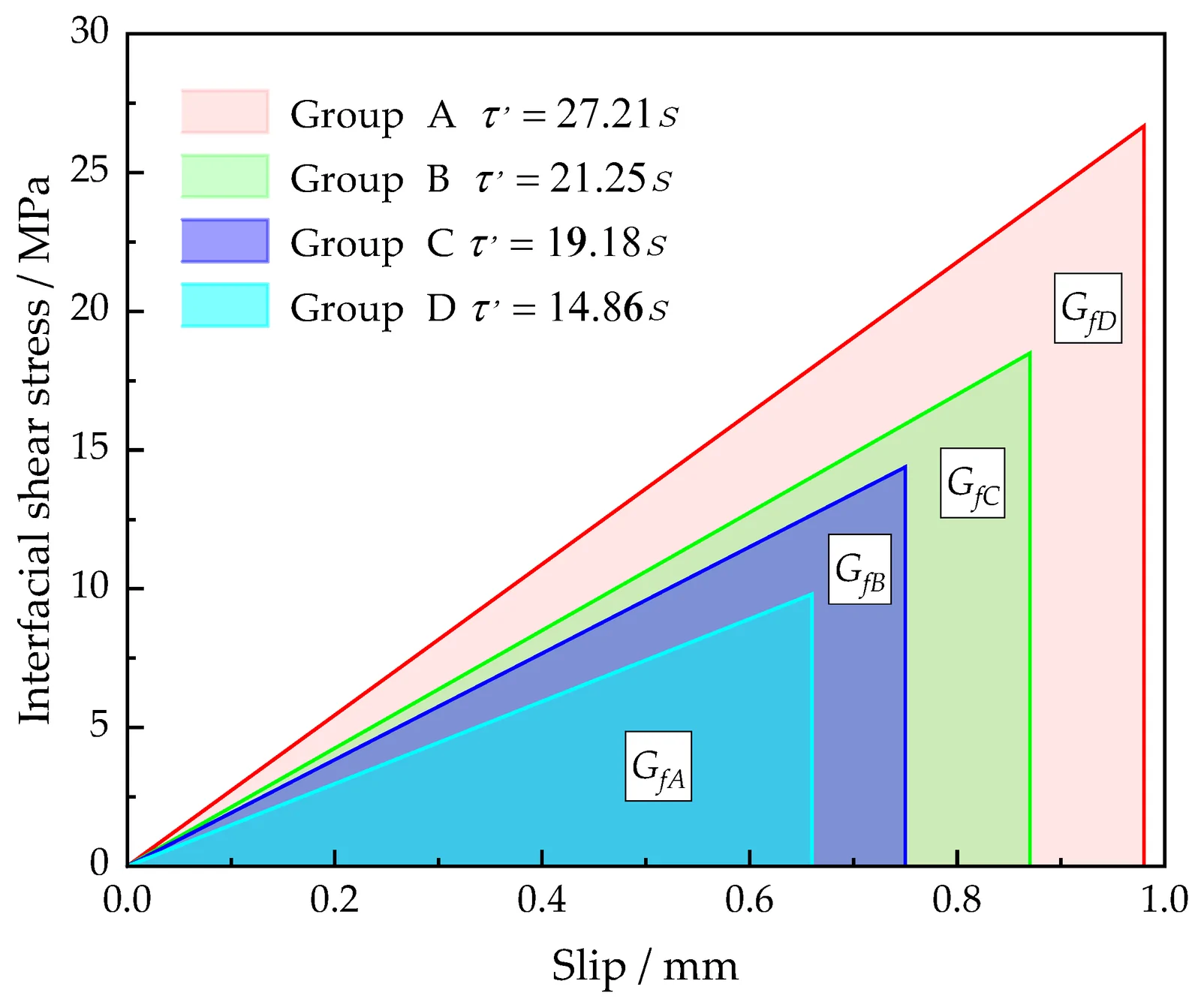
Bond–slip constitutive models of the specimens in each group.

The interfacial shear stress and slip exhibit an approximately linear relationship, which can be characterized by a bilinear constitutive model from Figure 9. Owing to the low ductility of the adhesive used, the specimens displayed pronounced brittle failure, and no softening stage was present in the interfacial shear stress. Consequently, the simplified bilinear model adopted in this study lacks the descending branch typically found in conventional bilinear models [1]. The prediction band at a 95% confidence interval for the simplified bilinear model covers most of the experimental data points, indicating that the model satisfactorily represents the bond–slip behavior of the specimens. Moreover, the agreement is better for Groups A and B than for Groups C and D. The bond–slip constitutive models for the CFRP-to-steel interface of each group of specimens are shown in Figure 10. The corresponding values of the maximum interfacial shear stress *τ*_max_ and maximum slip *δ*_max_ for each group are listed in Table 4. It can be observed from Table 4 that both the maximum interfacial shear stress *τ*_max_ and the maximum slip *δ*_max_ decrease with increasing adhesive layer thickness.

### 4.2. Ultimate Load Capacity

**Figure 11:**
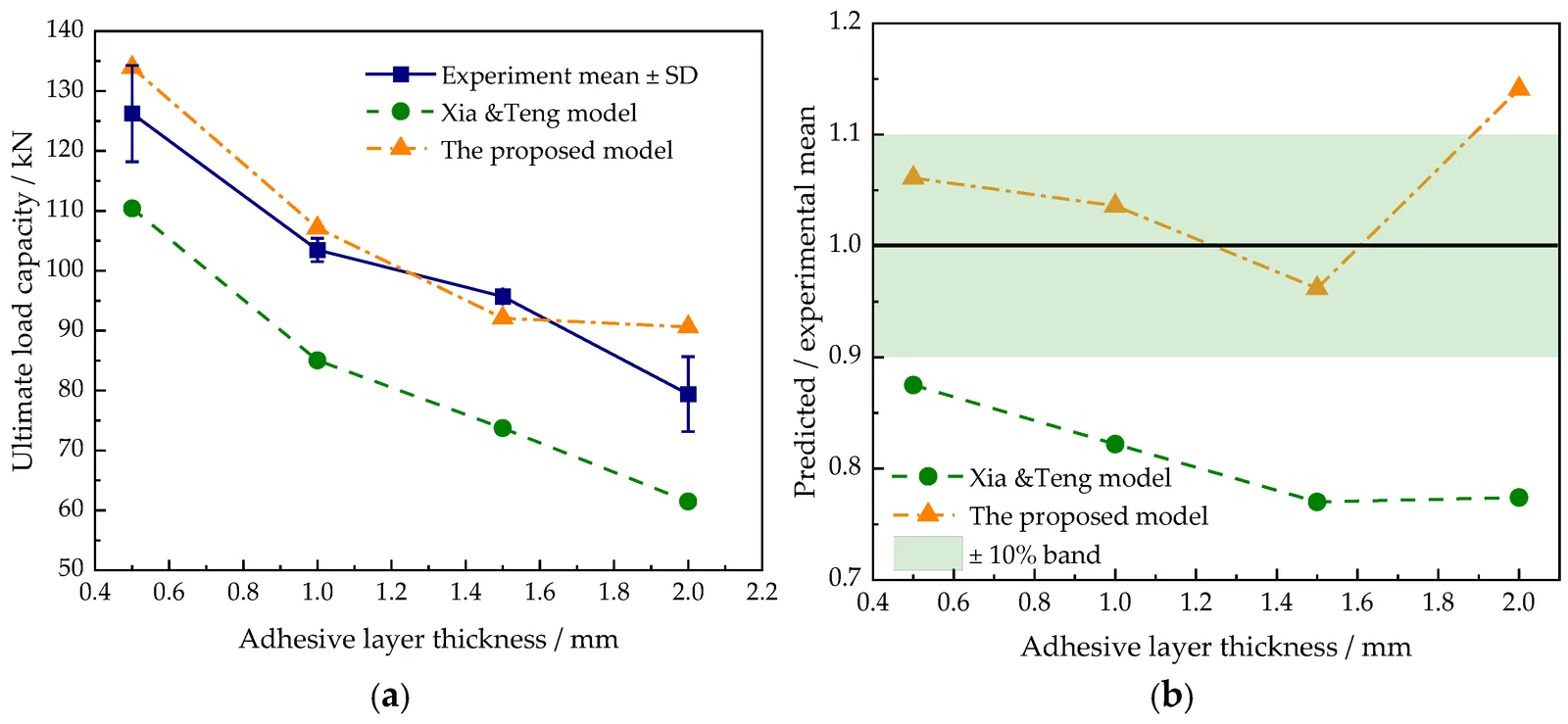
Comparison of the experimental and predicted ultimate load capacities: (**a**) Experimental group means ± sample SD and model predictions. (**b**) Predicted-to-experimental mean ratios with the ±10% band.

The ultimate load capacity of a CFRP-to-steel bonded joint is commonly estimated with the Xia & Teng model [20]:

(6) $$P_{\mathrm{ult}}=b_{c}\sqrt{2G_{f}E_{c}t_{c}}$$

where *G_f_* denotes the interfacial fracture energy, defined as the area under the bond–slip curve shown in Figure 11. The endpoint of the ascending branch corresponds to the maximum shear stress *τ*_max_ and the maximum slip *δ*_max_, the values of which are listed in Table 3. For the bilinear bond–slip constitutive model proposed in this study, the interfacial fracture energy *G_f_* is expressed as *G_f_* = 0.5*τ*_max_ · *δ*_max_. Substituting this expression into Equation (6) gives

(7) $$P_{\mathrm{ult}}=b_{c}\sqrt{\tau_{\max}\delta_{\max}E_{c}t_{c}}$$

In the bilinear bond–slip model proposed herein, the slope of the ascending branch is defined as the interfacial stiffness *K*, i.e., *K* = *τ*_max_/*δ*_max_. Substituting this relationship into Equation (7) and rearranging the terms yields

(8) $$P_{\mathrm{ult}}=b_{c}\tau_{\max}\sqrt{E_{c}t_{c}/K}$$

For the specimens with different adhesive layer thicknesses in this test, the interfacial stiffness *K* was found to be constant for each adhesive thickness group, as listed in Table 3. It can be seen from Table 3 that the interfacial stiffness *K* decreases with increasing adhesive layer thickness. Across the four groups, *K* decreased from 27.21 MPa/mm at *t_a_* = 0.5 mm to 14.86 MPa/mm at *t_a_* = 2.0 mm, a reduction of 45.4%. This monotonic decrease is consistent with the delayed engagement of the bonded length observed in Section 3.3 and shows that adhesive layer thickness affects the load-transfer stiffness rather than acting only through the adhesive tensile strength.

Regarding the maximum shear stress *τ*_max_, numerous researchers have conducted investigations. Xia & Teng [20] suggested that *τ*_max_ is related to the ultimate tensile strength of the adhesive *f*_*t*,*a*_, proposing *τ*_max_ = 0.8 *f*_*t*,*a*_. He et al. [21] experimentally concluded that *τ*_max_ = 0.5 *f*_*t*,*a*_. Fernando [22] recommended *τ*_max_ = 0.9 *f*_*t*,*a*_ through his study. A review of these references reveals that most existing studies employed a single adhesive layer thickness, varying only the type of adhesive material. Consequently, it is generally believed that *τ*_max_ is solely dependent on *f*_*t*,*a*_. However, the experimental results of the present study clearly indicate that *τ*_max_ is not only related to *f*_*t*,*a*_ but also to the adhesive layer thickness *t_a_*. A regression analysis of the test data yields $\tau_{\max}=0.4f_{t,a}t_{a}^{-0.5}$. Substituting this expression into Equation (8) and rearranging gives

(9) $$P_{\mathrm{ult}}=0.4b_{c}f_{t,a}t_{a}^{-0.5}\sqrt{E_{c}t_{c}/K}$$

For CFRP–steel double-strap specimens bonded with a low-ductility adhesive, a bilinear constitutive model without a softening stage can be adopted. The ultimate load capacity of the interface can be calculated using Equation (9). Equation (9) proposed in this paper is applicable to the adhesive type and adhesive layer thickness adopted in this study. The calculated results from the proposed model are summarized in Table 4. Table 4 also summarizes the mean values and standard deviations (SDs) of the ultimate load capacity for each specimen group. A comparison between the experimental and predicted ultimate load capacities is presented in Figure 11.

Figure 11 provides a visual interpretation of these statistics. Figure 11a compares the experimental group means, including their within-group standard deviations, with the two model predictions over the tested thickness range. It shows both the overall reduction in capacity with increasing adhesive layer thickness and the consistently lower predictions of the Xia & Teng model. The proposed model follows the experimental capacity level more closely over most of the range. Figure 11b plots the predicted-to-experimental mean ratio against adhesive layer thickness. The horizontal line at unity represents exact agreement, and the shaded band from 0.90 to 1.10 provides a direct visual reference for deviations within ±10%. All Xia & Teng predictions lie below this band or near its lower side, confirming a systematic negative bias. In contrast, the proposed model ratios for 0.5, 1.0, and 1.5 mm lie within the ±10% band, demonstrating improved calibration across these thicknesses. At ta = 2.0 mm, the proposed model overestimated the experimental mean by 14.1% and fell outside the ±10% band.

Two complementary error metrics were used to compare the models. The mean absolute percentage error (MAPE) measures the mean absolute prediction error relative to the experimental capacity and is therefore dimensionless. It allows errors to be compared across groups with different capacity levels. MAPE was calculated as

(10) $$\mathrm{MAPE}=\frac{1}{n}\sum_{i=1}^{n}\left|\frac{P_{i}^{\exp}-P_{i}^{\mathrm{pred}}}{P_{i}^{\exp}}\right|\times 100\%$$

where $P_{i}^{\exp}$ and $P_{i}^{\mathrm{pred}}$ are the experimental and predicted ultimate load capacities for thickness group *i*, and *n* = 4 is the number of groups. The comparison was performed at group level because the proposed model gives one prediction per thickness, whereas the Xia & Teng values were first averaged within each group. Both models were thus assessed against the same experimental quantity.

The root-mean-square error (RMSE) measures the typical absolute deviation between predicted and experimental capacities in kilonewtons. Squaring the residuals gives greater weight to large errors, making RMSE sensitive to isolated poor predictions. It was calculated as

(11) $$\mathrm{RMSE}=\sqrt{\frac{1}{n}\sum_{i=1}^{n}{\left(P_{i}^{\exp}-P_{i}^{\mathrm{pred}}\right)}^{2}}$$

where the symbols have the same meanings as in Equation (10). Lower values of both metrics indicate better agreement. MAPE describes relative accuracy, whereas RMSE retains the physical scale of the capacity error. Reporting both prevents a model from appearing accurate only because of the chosen units or the magnitude of a particular group.

As adhesive layer thickness increased from 0.5 to 2.0 mm, the experimental mean capacity decreased from 126.24 to 79.42 kN (37.1%). Both models reproduced the monotonic decline, but the Xia & Teng model systematically underestimated the capacity, with predicted-to-experimental ratios of 0.770–0.875, a mean absolute percentage error (MAPE) of 19.0%, and a root-mean-square error (RMSE) of 18.7 kN. In contrast, the proposed model yielded ratios of 0.962–1.141, a MAPE of 6.9%, and an RMSE of 7.3 kN. Incorporating adhesive layer thickness therefore reduced MAPE by 63.7% and RMSE by 61.1% relative to the Xia & Teng model for the present dataset. The main improvement is the removal of the systematic underprediction produced by a thickness-independent *τ*_max_ assumption.

## 5. Conclusions

This study systematically investigated the interfacial bond behavior of CFRP-to-steel joints through double-lap tensile–shear tests, with particular emphasis on the effect of adhesive layer thickness on ultimate load capacity. The conclusions presented below are limited to the adhesive used, the double-lap joint configuration, and the four adhesive layer thicknesses investigated. Their applicability to other adhesives, joint configurations, or thickness ranges requires further investigation. Based on the experimental results, the principal findings are as follows:

(1) Specimen failure occurred without obvious warning and was characterized as brittle failure. All specimens failed abruptly by mixed debonding at the CFRP–adhesive and steel–adhesive interfaces. Increasing adhesive layer thickness from 0.5 to 2.0 mm reduced the mean ultimate load capacity from 126.24 to 79.42 kN, a decrease of 37.1%. The corresponding mean nominal interfacial shear stress decreased from 8.42 to 5.29 MPa.
(2) At low load levels, the interfacial shear stress tended to decrease from the loaded end toward the fixed end. At higher load levels, the shear stress was relatively high at both ends and lower in the middle; moreover, under the same load level, the shear stress at the loaded end was greater than that at the fixed end. The CFRP plate was more actively engaged in load transfer at the loaded end than at the fixed end. Increasing adhesive layer thickness delayed engagement of the bonded length and reduced the fitted interfacial stiffness from 27.21 to 14.86 MPa/mm.
(3) The bond–slip response rose approximately linearly to abrupt failure and showed no resolved descending branch. The proposed capacity model links maximum interfacial shear stress to both adhesive tensile strength and layer thickness. Relative to the Xia and Teng model, it reduced group-level MAPE from 19.0% to 6.9% and RMSE from 18.7 to 7.3 kN.

## Figures and Tables

**Table 1:** Mechanical properties of materials.

Mechanical Properties	CFRP	Adhesive	Steel
Tensile strength (MPa)	2625.5	49.2	625.9
Elastic modulus (GPa)	180	4.5	209
Elongation (%)	1.7	/	23.2
Tensile–shear strength of steel-steel joints (MPa)	/	23	/

**Table 2:** Specimen geometry and measured adhesive layer thickness.

Specimen	Target Adhesive Layer Thickness (mm)	Measured Adhesive Thickness Value (mm)				
		*t*_1_	*t*_2_	*t*_3_	*t*_4_	Mean ± SD
A-0.5-1	0.5	0.472	0.508	0.486	0.494	0.490 ± 0.015
A-0.5-2	0.5	0.525	0.491	0.513	0.507	0.509 ± 0.014
A-0.5-3	0.5	0.463	0.487	0.479	0.495	0.481 ± 0.014
B-1.0-1	1.0	0.976	1.012	0.988	0.996	0.993 ± 0.015
B-1.0-2	1.0	1.041	0.997	1.023	1.015	1.019 ± 0.018
B-1.0-3	1.0	0.965	0.991	0.982	1.002	0.985 ± 0.016
C-1.5-1	1.5	1.493	1.527	1.506	1.514	1.510 ± 0.014
C-1.5-2	1.5	1.455	1.481	1.468	1.476	1.470 ± 0.011
C-1.5-3	1.5	1.512	1.538	1.525	1.533	1.527 ± 0.011
D-2.0-1	2.0	1.972	2.004	1.986	1.994	1.989 ± 0.014
D-2.0-2	2.0	2.055	2.011	2.039	2.027	2.033 ± 0.019
D-2.0-3	2.0	1.948	1.976	1.962	1.984	1.968 ± 0.016

**Table 3:** Test results of ultimate load capacity.

Specimen	Ultimate Load Capacity *P*_u_ (kN)	Mean Ultimate Load Capacity *P*_u_ (kN)	Mean Nominal Interfacial Shear Stress *τ*_nom_ (MPa)
A-0.5-1	133.91	126.24	8.42
A-0.5-2	117.89		
A-0.5-3	126.92		
B-1.0-1	101.54	103.47	6.90
B-1.0-2	105.42		
B-1.0-3	103.46		
C-1.5-1	96.38	95.72	6.38
C-1.5-2	95.07		
C-1.5-3	95.71		
D-2.0-1	84.20	79.42	5.29
D-2.0-2	72.33		
D-2.0-3	81.74		

**Table 4:** Theoretical results of ultimate load capacity.

Adhesive Layer Thickness *t*_a_ (mm)	The Proposed Bilinear Constitutive Model			Ultimate Load Capacity *P*_u_ (kN)				
	*τ*_max_ (MPa)	*δ*_max_ (mm)	*K* (MPa/mm)	Experiment Mean ± SD	Xia & Teng	Xia & Teng/Exp.	Proposed	Proposed/Exp.
0.5	26.67	0.98	27.21	126.24 ± 8.03	110.42	0.875	133.92	1.06
1.0	18.49	0.87	21.25	103.47 ± 1.94	85.04	0.822	107.16	1.04
1.5	14.39	0.75	19.18	95.72 ± 0.66	73.74	0.770	92.09	0.96
2.0	9.81	0.66	14.86	79.42 ± 6.26	61.49	0.774	90.61	1.14

## Data Availability

The original contributions presented in this study are included in the article. Further inquiries can be directed to the corresponding author.
